# Zebrafish in Inflammasome Research

**DOI:** 10.3390/cells8080901

**Published:** 2019-08-15

**Authors:** Gabriel Forn-Cuní, Annemarie H. Meijer, Monica Varela

**Affiliations:** Institute of Biology Leiden, Leiden University, Einsteinweg 55, 2333 CC Leiden, The Netherlands

**Keywords:** inflammasome, zebrafish, inflammatory caspases, gasdermin, evolutionary conservation, pyroptosis, intravital imaging, animal models

## Abstract

Inflammasomes are cytosolic multiprotein complexes that regulate inflammatory responses to danger stimuli and infection, and their dysregulation is associated with an increasing number of autoinflammatory diseases. In recent years, zebrafish models of human pathologies to study inflammasome function in vivo have started to emerge. Here, we discuss inflammasome research in zebrafish in light of current knowledge about mammalian inflammasomes. We summarize the evolutionary conservation of inflammasome components between zebrafish and mammals, highlighting the similarities and possible divergence in functions of these components. We present new insights into the evolution of the caspase-1 family in the teleost lineage, and how its evolutionary origin may help contextualize its functions. We also review existing infectious and non-infectious models in zebrafish in which inflammasomes have been directly implicated. Finally, we discuss the advantages of zebrafish larvae for intravital imaging of inflammasome activation and summarize available tools that will help to advance inflammasome research.

## 1. Introduction

Inflammasomes are cytosolic protein complexes that regulate the activation of the immune response to cellular stress associated with microbial infections and tissue damage [1]. Activation of inflammasome complexes can lead to secretion of pro-inflammatory cytokines such us interleukin-1β (IL-1β) and/or pyroptosis, a regulated form of lytic cell death that is accompanied by the release of cellular content into the environment [2,3,4,5]. Dysregulation of inflammasome activation has been linked with a vast number of inflammatory pathologies, including inflammatory bowel disorders, such as Crohn’s disease [6], autoimmune diseases, such as systemic lupus erythematosus [7], and neurodegenerative diseases, such as Alzheimer’s and multiple sclerosis [8]. Recent research also highlights the importance of inflammasome signaling beyond immunity, for example in regulating metabolic processes such as glycolysis, eicosanoid synthesis, lipid metabolism, and autophagy [9].

Inflammasome activation is usually classified as either canonical or non-canonical, depending on the activation pathways and protein components assembled. The canonical inflammasome pathway requires cytosolic sensors for detecting pathogen-associated or danger-associated molecular patterns (PAMPs or DAMPs, respectively). These sensors are pattern recognition receptors (PPRs) from the Nod-like receptor and LRR-containing receptor (NLR) and AIM2-like receptor (ALR) families, or proteins containing TRIM motifs, like pyrin [10]. Upon stimulation, sensors recruit the adaptor protein ASC (apoptosis-associated speck-like protein, also known as pycard) for the formation of a multimeric protein complex where the pro-inflammatory caspase-1 (CASP1) is recruited and activated [11]. Enzymatically active CASP1 cleaves the pro-inflammatory downstream effectors IL-1β and interleukin 18 (IL-18) into their bioactive forms [11].

In the non-canonical inflammasome pathway, caspase-4 (CASP4) and caspase-5 (CASP5)- or its ortholog in mice, caspase-11 (CASP11)- are activated by direct binding to lipopolysaccharide (LPS), endotoxin derived from the outer membrane of Gram-negative bacteria [12], or cell endogenous oxidized lipids (oxPAC) [13]. However, it was recently revealed that CASP11 can also be recruited to the ASC-dependent NLRP6 inflammasome, therefore getting activated in a canonical way and blurring the difference between these categories [14]. Activated CASP1 and CASP11 proteolytically cleave naturally autoinhibited gasdermin-D (GSDMD) into a C- and a N-terminal fragment [15,16], allowing the insertion of N-terminal fragments into the plasma membrane for formation of pores, induction of pyroptotic cell death and release of cellular content into the extracellular space [17].

To further advance our understanding of the in vivo regulation, activation, and function of inflammasome complexes, animal models are required. Over the last decades, the zebrafish has consolidated its position as a powerful model to explore vertebrate immunity and dissect the dynamics of components at the host-pathogen interface of microbial infections [18,19,20,21]. Characteristics traditionally paired to invertebrate models such as ease of effective genetic manipulation, fast life cycle, small size, virtually infinite specimens, suitability for high-throughput, and unparalleled in vivo intravital microscopy due to embryo transparency can be exploited in a vertebrate model with impactful translational potential to the clinic [22]. To date, zebrafish models have already greatly contributed to our knowledge of human infections that are difficult to replicate in mammalian hosts in vivo, such as tuberculosis [23].

As a result, zebrafish are now increasingly used to study functions and evolution of inflammasomes [24,25,26]. The optical transparency of zebrafish early life stages provides unprecedented possibilities to study inflammasome activation and dynamics in vivo [27]. Furthermore, studies in zebrafish models have already contributed insights into the regulation of the inflammasome, not only during infections, inflammatory processes, and disease, but also during cellular homeostasis [27,28,29,30,31]. However, the translational potential from zebrafish to humans in this research is subject to the different host environments that are the result of 450 million years of evolutionary divergence. For appropriate extrapolation of findings, it is important to understand homologies between zebrafish, mammalian models, and the human situation. In this review, we discuss current knowledge of inflammasomes in zebrafish and present new insights into the evolution of the caspase-1 family.

## 2. Evolutionary Conservation of Inflammasome Components in Zebrafish

In zebrafish, both canonical and non-canonical inflammasome activation mechanisms have been described, although specific components and activation dynamics differ from mammalian inflammasomes. While intracellular pathway and signaling components are highly conserved in vertebrate evolution, components exposed to the host-pathogen interface, such as inflammasome sensors and effectors are under strong evolutionary pressure and are therefore more divergent [32,33]. Here, we will go through different zebrafish inflammasome components and how they compare to the ones known in mammalian inflammasomes. A summary of some assays and tools currently available for inflammasome research in zebrafish are listed in Table 1 and Table 2, respectively.

### 2.1. Inflammasome Sensors

**Figure 1:**
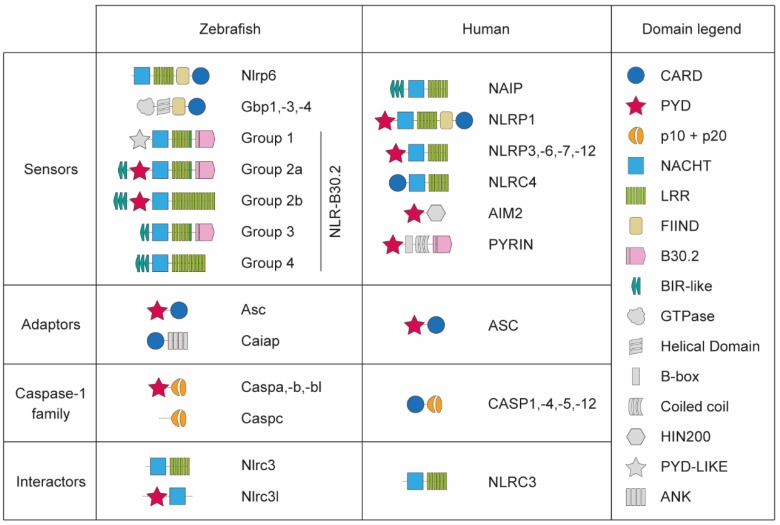
Schematic representation of protein domains in the components involved in inflammasome function in zebrafish.

The NLR repertoire in zebrafish is significantly different to that in mammals, including cases of both divergent and convergent evolution (see [54] for a recent in-depth review). In humans and mice, the proteins of the NLR-family NAIP, NLRC4, NLRP1, NLRP3, NLRP6, NLRP7, and NLRP12 have been either suggested or confirmed to act as sensors and form functional inflammasomes [11]. Despite the fact that other genes of the NLR-family are conserved in one-to-one orthologs between zebrafish and mammals (including NOD1/NLRC1, NOD2/NLRC2, NLRC3, and NLCR5), most human NLRs involved in inflammasomes do not have one-to-one orthologues in zebrafish [55] (Figure 1). In contrast, the zebrafish genome contains more than 400 NLR genes, most of them evolved from the specific NLR-B30.2 gene subfamily [55]. So far, two NLRP subfamily proteins have been reported to be involved in inflammasomes in zebrafish. One of the proteins referred to as Nlrp6 has been proposed as a homolog of NLRP1 despite lacking the N-terminal PYRIN (PYD) domain [56]. Nlrp6 is able to form inflammasome complexes in response to *Edwarsiella piscicida in vivo,* confirming its function as inflammasome sensor [56]. Another predicted protein, referred to as Nlp3 isoform X1, is able to interact with zebrafish Asc in vitro via the interaction of their PYD domains [57]. However, the downstream caspase recruitment and activation dynamics differ to the ones in the mammalian NLRP inflammasomes. Therefore, the evolutionary relationship between these proteins and the mammalian NLRPs is still not resolved. It remains to be determined which role the many other NLR-family proteins of zebrafish play in inflammasome activation.

In addition to NLR-family sensors, the interferon-induced AIM2 and Pyrin are also involved in mammalian inflammasome assembly [10]. The genes coding for these proteins emerged after the fish-tetrapod divergence and thus are not present in zebrafish [52,55]. However, interferon-induced proteins are involved in inflammasome assembly in zebrafish, as in mammals. The zebrafish interferon-induced guanylate-binding proteins (GBPs) Gbp1, Gbp3, and Gbp4 contain additional FIIND and C-terminal CARD domains that strikingly resemble those of NLRP1, hinting that they may fill the NLRP-role to assemble inflammasome complexes [54,58,59] (Figure 1). GBPs are also involved in the modulation of inflammasome assembly in mammals [58,60,61], although their specific role is still not clear. In zebrafish, Gbp4 has been shown to be required to form a functional inflammasome involved in the clearance of *Salmonella enterica* serovar Typhimurium in an Asc-dependent manner in neutrophils [39]. The activation of this inflammasome does not lead to cleavage and release of Il1b and pyroptosis, but instead results in the processing of prostaglandin D2 by a proinflammatory caspase [39]. This mechanism could therefore be similar to the induction of eicosanoid synthesis downstream of caspase-1 activation reported in mammalian inflammasomes [9,62].

### 2.2. Inflammasome Adaptors

After the recognition of their ligand, sensor proteins can recruit the adaptor protein ASC. The ASC protein has a C-terminal CARD and a N-terminal PYD domain, allowing it to interact with sensor proteins with the PYD domain and with pro-inflammatory caspases with its CARD domain [63] (Figure 1). Upon recruitment, ASC self-assembles into large signalling platforms, named ASC specks or foci, which are considered a hallmark of canonical inflammasome activation [64,65]. Unlike in mammals, zebrafish Asc PYD domain is critical for both auto-assembly and downstream proinflammatory caspase recruitment via PYD-PYD interactions [27,57]. Despite this difference, zebrafish Asc functionality seems to be conserved between zebrafish and mammals, as pyroptosis of zebrafish keratinocytes and macrophages has been recently visualized after Asc speck formation in vivo [27,29].

In addition to Asc, an ankyrin domain-containing protein named Caiap has been reported as a new inflammasome adaptor in zebrafish [40]. Caiap, which does not have a human or mouse homolog, acts in macrophages downstream of flagellin recognition and self-oligomerizes via its CARD domains after directly interacting with active caspases [25,40] (Figure 1). However, Caiap has not been shown to interact with sensor proteins yet, thus requiring already active caspase to further promote inflammasome function.

### 2.3. Zebrafish Pro-Inflammatory Caspases

As in humans, there are four proinflammatory caspase-1-like genes annotated in the current zebrafish genome version GRCz11; *caspa* (also known as *caspy*), *caspb* (also known as *caspy2*), *caspbl*, and *caspc*. However, there is a striking difference between zebrafish and mammalian proinflammatory caspases; while all human and mouse proinflammatory caspases have a CARD prodomain, *caspa*, *caspb*, and *caspbl* have a PYD prodomain, and *caspc* does not have any prodomain [66] (Figure 1). It has been assumed in a number of studies that *caspa* is a functional homolog of mammalian *CASP1* and that *caspb* is a functional homolog of mammalian *CASP4/5/11* [25,27,39,41,42,52]. This assumption was originally backed by percentage of protein sequence homology and protease substrate preference [41]. Further functional analysis reported that only the Caspa PYD domain, but not the one of Caspb, is able to directly interact with Asc [27,41,57], and therefore promote Asc-dependent pyroptosis [27]. On the other hand, the Caspb PYD prodomain, but not the one in Caspa, is able to bind to LPS when overexpressed in HEK293T cells [42]. Thus, these studies showed that Caspa promotes pyroptosis in a canonical way, similar to mammalian CASP1, while Caspb mediates non-canonical pyroptosis, like mammalian CASP4/5/11.

However, there are intriguing contradictions to the assumption that Caspa and Caspb would be equivalent to CASP1 and CASP4/5/11, respectively. While the abilities of Caspa and Caspb to cleave Gasdermin remains to be studied, they are both able to cleave Il1b, albeit with different specificities [52]. Interestingly, Caspa and Caspb can directly interact in vitro via its PYD domains, thus opening the possibility of Caspa/Caspb oligomerization needed to fully mature Il1b [57]. In contrast, mammalian CASP4/5/11 cleave Gasdermin D but not proinflammatory cytokines during pyroptosis [67]. It has also been reported that despite not being able to directly interact with Asc via PYD-PYD interactions, Caspb is recruited and activated in Asc specks [56]. Moreover, we have recently shown that Caspa can be activated through Asc-dependent and Asc-independent mechanisms in vivo during *M. marinum* infection, and its knockdown phenotype can be rescued by overexpression of mouse *Casp11* but not *Casp1* [29]. The results suggest that, while Caspb has been shown to function equivalently to CASP4/5/11 in LPS-triggered ASC-independent activation, Caspa performs the same role in another ASC-independent inflammasome.

**Figure 2:**
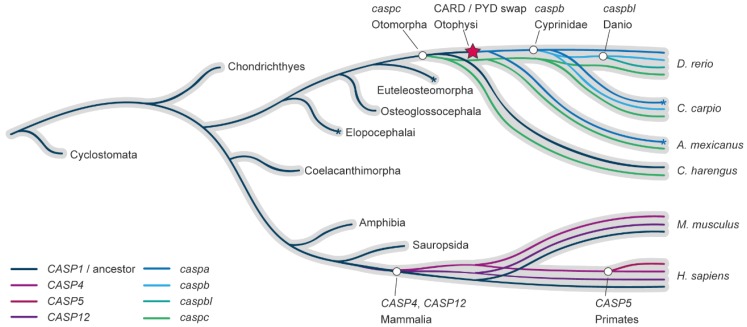
Evolutionary model of the caspase-1-like family in fish and mammals. Reconciliation of the caspase-1-like gene subfamily evolution inside of the species evolution (grey). Gene duplication events are depicted with a white circle symbol, indicating in the label the last common ancestor in which the duplication has been found. The CARD/PYD prodomain swap is indicated with a red star. Asterisks indicate gene expansions in lineages outside of the scope of this study.

In order to clarify the relationship between mammalian and zebrafish genes involved in inflammasome complexes, we reconstructed the caspase-1-like gene family evolution in vertebrates from lampreys to humans, with specific interest in the zebrafish lineage. Contrasting to previous studies, we focused our evolutionary analysis on the conserved C-terminal p10 and p20 catalytic domains, thus avoiding skewed results based on the homology of the differential or absent prodomain motifs. The methods, in-depth reconstruction of the pro-inflammatory caspase-1 family evolution in the fish lineage, and the consequences that this evolutionary model has in the understanding of the evolution of this family in fish can be found in Supplementary Materials. Importantly, both maximum likelihood and Bayesian inference methodologies produced similar tree topologies that strongly agree in that all zebrafish caspase-1-like genes are derived from a common ancestor in the fish lineage after the fish-tetrapod divergence. That is, the proinflammatory caspase-1 family duplications in zebrafish arose in the fish lineage, independently of the mammalian evolution (Figure 2).

In consequence, the four zebrafish proinflammatory caspases, *caspa*, *caspb*, *caspbl*, and *caspc* are many-to-many homologs to the mammalian *CASP1*, *CASP4*/5/11, and *CASP12*, the lesser known fourth member of the pro-inflammatory caspase family in mammals. Based on this evolutionary relationship, different activation patterns and regulation, subfunctionalization, duplicate or even new functions are expected between Caspa, Caspb, Caspbl, and Caspc, and the range of functional homology to mammalian caspase-1-like that may exist will be determined by the environment, for example being specific for different cell types, processes, or infections.

### 2.4. Inflammasome Effectors

As with sensors, effector components downstream of inflammasome assembly also present high divergence to their mammalian counterparts. For example, the IL-1 family in fish is highly diversified [26]. To date, there are no reports of a functionally homolog IL-1α in fish and, while present in some teleosts, the IL-18 gene seems to have been lost in the zebrafish lineage [24,68,69]. However, zebrafish possess a functional homolog of mammalian IL-1β, which is cleaved by enzymatically active proinflammatory caspases [26,52].

In the zebrafish genome (version GRCz11) three copies of genes encoding proteins with a Gasdermin pore forming domain are annotated: *pjvk*, *gsdmea* and *gsdmeb*. In silico analysis of protein sequences predicted that Gsdmeb has a Caspase-1 cleavage site, while Gsdmea is predicted to be cleaved by Caspase-3 [29]. Further functional analysis has confirmed that inhibition of *gsdmea* can partially inhibit cell death in microglia in a temperature sensitive *puer* zebrafish mutant harbouring a loss-of-function mutation in the *nlrc3-like* receptor [30]. Furthermore, we have shown that knockdown of *gsdmeb* can inhibit pyroptosis in vivo in a zebrafish model of TB [29]. Therefore, in this infection model, Gsdmeb emerges as the equivalent of mammalian GSDMD.

## 3. Zebrafish Models of Inflammasome Regulation in Health and Disease

### 3.1. Inflammasome Function in Zebrafish Infectious Models

**Figure 3:**
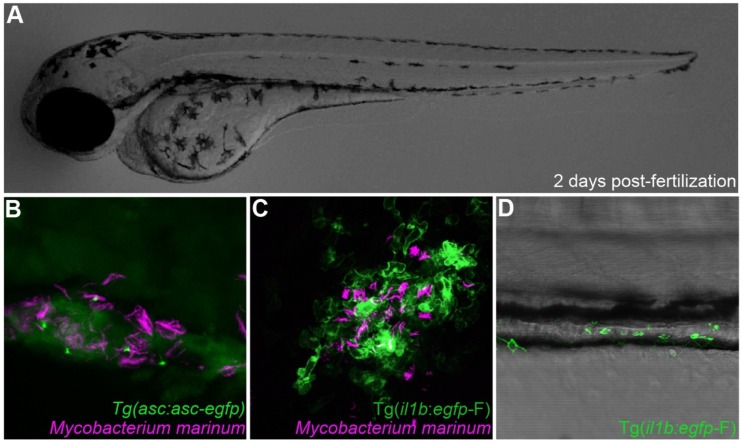
Inflammasome components can be directly visualized in zebrafish. (**A**) Transparent 2 days post-fertilization zebrafish larvae were challenged by previously described infection and inflammation conditions [29,70]. (**B**) Visualization of Asc speck formation in zebrafish macrophages infected with *Mycobacterium marinum*. (**C**) *il1b* reporter gene visualization upon *Mycobacterium marinum* infection. (**D**) *il1b*-producing leukocytes are recruited to the intestine of a zebrafish larvae in a model of Copper-induced intestinal inflammation.

The possibility of following infections in real time at a cellular level in the context of a whole individual makes the zebrafish a versatile model for studying inflammasome function in interaction with pathogens while maintaining the context of a complex population of cells (Figure 3). While the dynamics of the Il1b response as an inflammatory marker has been studied in several infectious diseases modelled in zebrafish [34,47,48,49,50,51,52], research focusing on inflammasome assembly and function using zebrafish is only starting to emerge (Table 3).

Importantly, the use of zebrafish has unraveled the implication of different cell types (macrophages vs. neutrophils) and their inflammasomes in relevant human pathologies caused by bacterial species like *Listeria monocytogenes* [28] and *Salmonella enterica* serovar Typhimurium [39]. The role of neutrophils in clearing *Listeria monocytogenes* has been historically unclear, but studies performed in zebrafish have proved that neutrophils are important to control the infection downstream of inflammasome activation, which depends mainly on macrophages [28]. In contrast, the eicosanoid synthesis that is promoted after inflammasome activation in neutrophils, but not macrophages, is crucial for the clearance of *Salmonella enterica* serovar Typhimurium [39].

The infectious disease most extensively studied in zebrafish is tuberculosis (TB) [23,71,72]. Studies in zebrafish using the natural fish pathogen *Mycobacterium marinum*, a close relative to causative agent of TB, *Mycobacterium tuberculosis*, have been crucial for a better understanding of the pathogenesis of mycobacterial infection and the biology of granulomas, the hallmark inflammation foci formed in TB [73,74]. Our recent study of inflammasome function during TB pathogenesis in the zebrafish model has demonstrated the role of zebrafish Caspa-dependent pyroptosis in granuloma expansion and pathogen dissemination [29]. Moreover, it has also been shown that the early neutrophil response during mycobacterial infection is inflammasome-dependent in this model [36].

Zebrafish inflammasomes have also been studied in the infection course of pathogens with high impact on aquaculture. In the zebrafish–*Edwarsiella piscicida* infection model, bacterial haemolysin can activate a Caspb-dependent pyroptotic activity in non-phagocyte cells [75], and an Asc-independent activation of Caspb, but not Caspa, is important for an efficient host defense [42].

Compared to our knowledge of bacterial diseases, inflammasome activation upon viral infections is less well understood. However, there is a central role of inflammasomes in pathogenesis and defense against viral infections [76,77]. Using spring viraemia of carp virus (SVCV) we could visualize viral-induced pyroptosis at a cellular level for the first time in the context of a whole organism [34]. Several viral infections have been successfully modelled in zebrafish [78] and further studies about the role of viral components in inflammasome activation using these models might shed light into new ways to treat viral diseases in the close future.

Importantly, research in zebrafish inflammasome function has been key to describing new inflammasome components, like the previously mentioned adaptor Caiap [40]. While Caiap is absent in placental mammals, studying the inflammasome function in zebrafish against *Salmonella enterica* serovar Typhimurium has also highlighted the importance of the evolutionary conserved WD repeat containing protein 90 (Wdr90) as a new inflammasome component [44]. Wdr90, of unknown function until the previously mentioned study, is induced by Gbp4 in zebrafish and mediates the cellular distribution of human NLRC4, but not NLRP3 or AIM2, while overexpressed in human cells [44]. Zebrafish infection with *Shigella flexneri* results in leukocyte cell death that resembles pyroptosis of human macrophages infected with *Shigella* [35]. Further research in this infection model has uncovered a new role of *sept7* and *sept15*, homologs of human *SEPT7* which is essential for septin filament assembly and function, in restricting Caspase-1 activation and pyroptosis during *Shigella* infection [38], opening the possibility of targeting these cytoskeleton components as a novel anti-inflammatory strategy.

### 3.2. Inflammasome Function in Zebrafish Non-infectious Models

There is strong evidence of the role of inflammasomes also in non-infectious pathologies (Table 3), such as chronic inflammatory conditions or wound healing [82,83]. One of the most common zebrafish models is the tailfin injury model, which involves the transection of zebrafish tail fin [84]. This stimulus creates a local inflammatory and wound healing response that culminates in complete fin regeneration over the course of 2–4 days [85]. The inflammatory response originated after a tail fin injury has been shown to be inflammasome-dependent [51]. In addition to understanding the outcome of inflammasome activation and how inflammasomes interact with other factors of the inflammatory response [47], the tail injury model could be useful to better understand the role of inflammasomes in tissue regeneration.

Obesity and diet-induced chronic inflammation is a rising epidemic with high risk to human health worldwide. This has motivated the creation of several models of induced and genetically-caused hepatic and intestinal inflammation in zebrafish [79,86,87]. Zebrafish exposed to high cholesterol diets suffer from acute inflammasome activation in intestinal epithelial cells resulting in an accumulation of inflammatory leukocytes around the intestine [45]. Already existing zebrafish models of chronic or autoinflammatory disorders might be useful to study the inflammasome role in the pathogenesis of these diseases in vivo.

In addition to autoimmune and inflammatory pathologies, inflammasomes are involved in the correct development and differentiation of cell populations in homeostasis. Recently, inflammasomes appeared to be intrinsically required for myeloid differentiation of hematopoietic stem and progenitor cells in zebrafish via GATA1 cleavage [31]. This function is conserved in mice models, and thus might potentially be an interesting target in the treatment of human inflammatory diseases in which altered hematopoiesis is associated [31,88]. Correct regulation of inflammasome function is also critical for homeostasis of tissue-resident macrophages of the central nervous system, known as microglia. The development and maintenance of the microglia population in zebrafish is dependent on the interaction between anti-inflammatory Nlrc3-like and Asc, thus preventing inappropriate macrophage activation and microglia death [30,81].

## 4. Concluding Remarks and Future Considerations

The use of zebrafish offers a solid, fast and cost-effective alternative to the use of mice as animal models to study development, innate immunity and infectious diseases [32,89]. It is therefore not a surprise that zebrafish models dedicated to investigating inflammasome function are starting to emerge. As we have reviewed, these models are already helping advance our understanding of the roles of inflammasomes in host-pathogen interactions [25,28,29,38], as well as during development and aseptic inflammation [27,31,81] and, in the future, they could help answer fundamental questions about the cellular context during in vivo inflammasome activation. They also open the possibility to develop high-throughput, high-content screenings to discover new modulators of inflammasome activity for therapeutic application.

However, the translational potential of findings in zebrafish models is constrained by the evolutionary divergence of some inflammasome components between teleosts and mammals. In that sense, the range of functional homology between zebrafish and human inflammasomes may be dependent on the specific environment modelled, as is the case for proinflammatory caspases. In any case, despite of the lack of one-to-one homology in most inflammasome components, the mechanism and functions of zebrafish and mammalian inflammasomes seem to be conserved: large multimeric proinflammatory complexes are assembled in response to recognition of specific ligands from sensor proteins of the NLR and interferon-stimulated families, inflammasomes can be activated by Asc-dependent signaling as well as in an Asc-independent manner via direct sensing of LPS, they promote pyroptosis via the cleavage of pore-forming proteins, and they are involved in maturation and release of proinflammatory cytokines and eicosanoids.

Thus, there is great potential for the use of zebrafish in inflammasome research, a rising field with many open questions, like the fate of ASC specks after pyroptosis, that could be answered using already available zebrafish tools in combination with advanced microscopy techniques in which this model really shines. In addition to the possibility of establishing new zebrafish disease models with the specific objective of advancing inflammasome research, several already well-established zebrafish infectious models are available that could be exploited for inflammasome research. This is for example the case for *Staphylococcus aureus* [79] and *Escherichia Coli* [48] infections, in which inflammasome activation is important for their pathogenesis and might be explored in the zebrafish animal model in the coming years.

## Figures and Tables

**Table 1:** General assays available for inflammasome research in zebrafish.

Readout	Assay	References
Cell death	TUNEL	[29,34,35]
	Sytox Green	[36]
Caspase activity	*in situ* caspase activity (FLICA)	[29,37,38]
	Fluorometric assay (e.g., Z-YVAD-AFC)	[28,29,39,40,41,42,43,44]

**Table 2:** Specific zebrafish tools available for inflammasome research.

Inflammasome Target/Aim.	Tool/Assay	References
Asc	Tg(*asc:asc-egfp*)	[27,29]
	Tg(*HSE:asc-mkate2*)	[27]
	*asc* CRISPR mutant	[30]
	*asc* TALEN mutant	[37]
	*asc* Translation-blocking MO	[27,29,39,40,45]
	*asc* Splice-blocking MO	[39,45,46]
	Asc zebrafish antibody	[27]
Caspa	*caspa* CRISPR mutant	[27,29]
	*caspa* Translation-bloking MO	[28,29,41]
Caspb	*caspb* Splice-blocking MO	[29,42]
	*caspb* CRISP	[36]
Il1b	TgBAC(*il1b:egfp*)	[47]
	Tg(*il1b:efgp*-F)	[48,49,50]
	*il1b* CRISPR mutant	[47]
	*il1b* Splice-blocking mo	[29,39,45,47,48,50,51]
	Il1b antibody	[34,52]
	Il1b antibody	[53]
Gsdmea	*gsdmea* Splice-blocking MO	[30]
Gsdmeb	*gsdmeb* Translation-blocking MO	[30]
	*gsdmeb* Splice-blocking MO	[29]

**Table 3:** Infectious and non-infectious zebrafish models in which inflammasome function has been reported.

	Model	References
Infectious	*Aeromonas hydrophila*	[49]
	*Burkholderia cenocepacia*	[50]
	*Edwarsiella piscicida*	[42,75]
	*Escherichia coli*	[48]
	*Francisella noatunensis*	[52]
	*Listeria monocytogenes*	[28]
	*Mycobacterium marinum*	[29,36,47]
	*Salmonella enterica* serovar Typhimurium	[39,40,44]
	*Shigella flexneri*	[35,38]
	*Staphylococcus aureus*	[79]
	Spring viraemia of carp virus	[34]
Non-infectious	Hematopoiesis	[31]
	Hepatic inflammation	[80]
	Intestinal inflammation	[45]
	Microglia homeostasis	[30,81]
	Tail fin transection	[51]

## Supplementary Materials

The following are available online at https://www.mdpi.com/2073-4409/8/8/901/s1, Figure S1: The consensus gene tree calculated by jPrime and MrBayes. Node labels indicate the % of trees in which the node is conserved., Figure S2: Maximum Likelihood gene tree calculated by Phyml. Node labels indicate statistical confidence. Figure S3: A) Conserved synteny of the *caspc* and B) the *pycard*/*caspa* locus in the Otomorpha lineage. **C**) Domain analysis of the caspase-1-like genes in the Otomorpha lineage. The CARD and PYD prodomains, and the catalytic domains are depicted with a blue circle, a red star, and a grey hexagon, respectively.
