# Supplementary material for: Zebrafish in Inflammasome Research
Source: Cells. 2019 Aug 15;8(8):901. doi: 10.3390/cells8080901 (PMC6721725; doi:10.3390/cells8080901)
Supplement: Supplementary file 1 [file cells-08-00901-s001.zip › cells-573843-SI.pdf]

Supplementary Materials

Zebrafish in Inflammasome Research

Gabriel Forn-Cuní, Annemarie H. Meijer and Monica Varela *

Institute of Biology Leiden, Leiden University, Einsteinweg 55, 2333 CC Leiden, The Netherlands
* Correspondence: m.varela.alvarez@biology.leidenuniv.nl

Received: 30 July 2019; Accepted: 13 August 2019; Published: date

Reconstruction of the Proinflammatory Caspase-1-like Family Evolution

An exhaustive tblastn search was performed against evolutionary-relevant genomes with the human *CASP1* and zebrafish *caspa*, *caspb*, *caspbl*, and *caspc* sequences. We included sequences from a total of 23 genomes: *Anguilla anguilla, Anolis carolinensis, Astyanax mexicanus, Callorhinchus milii, Clupea harengus, Cyprinus carpio, Danio rerio, Electrophorus electricus, Gallus gallus, Homo sapiens, Latimeria chalumnae, Lepisosteus oculatus, Lethenteron camtschaticum, Leucoraja erinacea, Mus musculus, Oreochromis niloticus, Petromyzon marinus, Poecilia formosa, Rhincodon typus, Scleropages formosus, Takifugu rubripes, Xenopus tropicalis,* and *Xiphophorus maculatus*. In some case, the target region was reannotated with GENSCAN webserver due to the lack of annotation of the region [1].

Protein domains were determined with the NCBI Conserved Domain Database [2], and/or the EXPASY PROSITE service [3]. To prevent clustering of the sequences based on their prodomain, only the core C20 and C10 region was aligned. The alignment of the 37 protein sequences was performed in the MAFFT server [4]. Ambiguously aligned columns were pruned with Gblocks server [5]. The best-fit model of amino acid replacement (JTT) was selected according to the Akaike Information Criterion (AIC) in ProtTest 3.2 [6]. To identify gene duplications and loss events during the evolution of the *CASP1* gene family, a reconciliation [7] of the obtained gene tree with the species evolution was performed. The divergence times among species were retrieved from the TimeTree database [8]. The gene family tree was estimated with jPrime 0.3.6 [9], in which four independent MCMC runs, each consisting of 1,000,000 iterations, were sampled once every 200 iterations. After discarding the first 500 samples for each run as burn-in, the final gene tree was obtained as a weighted consensus majority-rule tree from the four runs with MrBayes 3.2.6 [10] (Figure S1). We also calculated the maximum likelihood phylogenetic tree using PhyML v.20160207 [11] (Figure S2). The unrooted gene trees were rooted *a posteriori* based on the evolutionary knowledge that lampreys were the earliest species to diverge from the rest of vertebrates in this study. Interestingly, only one caspase-1-like sequence is present in their genome, hinting that all caspase-1 duplications occurred after their divergence. The most parsimonious reconciliation of the estimated gene tree and the species tree was performed with Notung 2.9 [12], represented with FigTree v1.3.1 and PrimeTV [13], and edited in Adobe Illustrator.

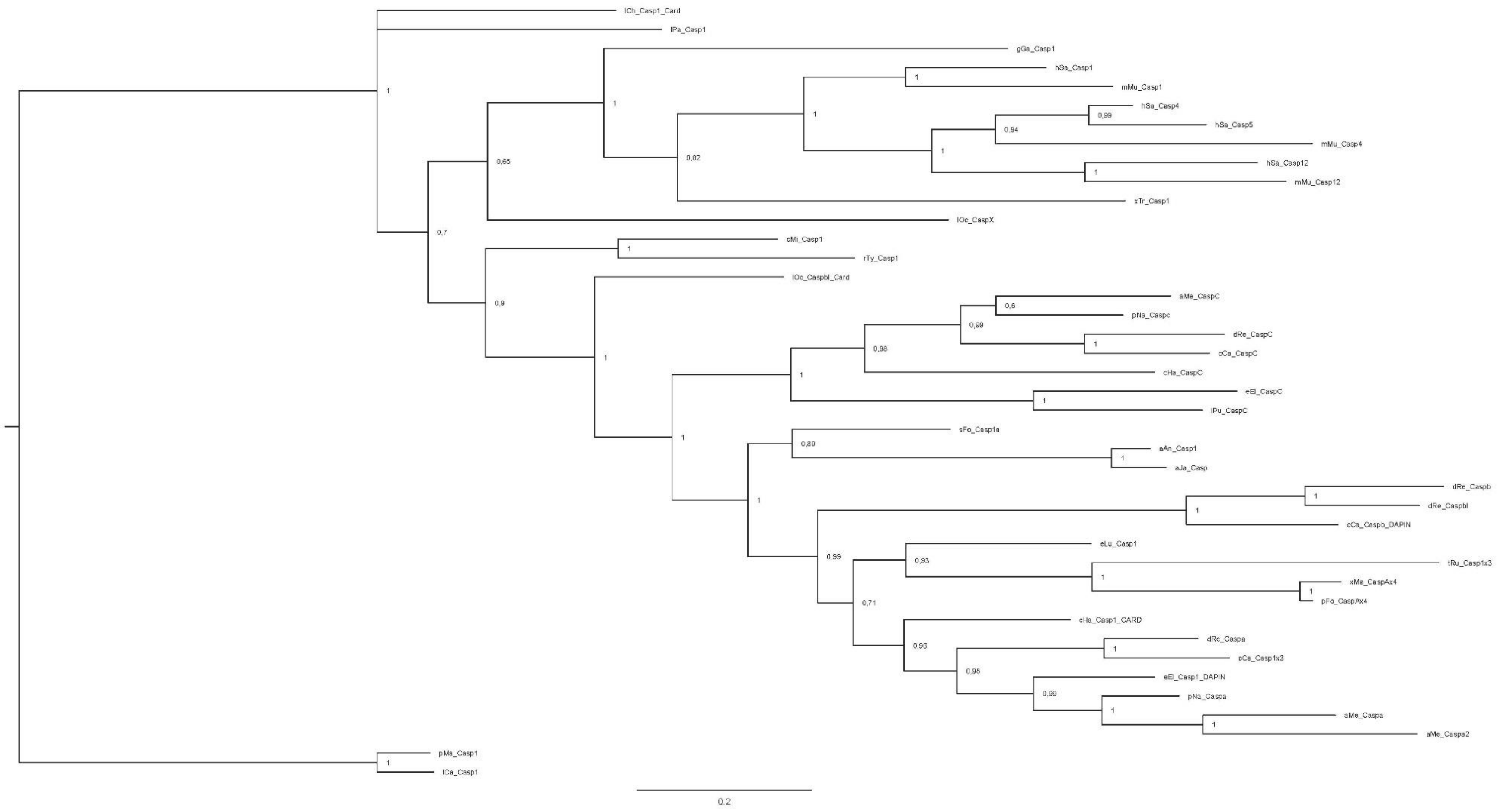

Figure 1. The consensus gene tree calculated by jPrime and MrBayes. Node labels indicate the % of trees in which the node is conserved.

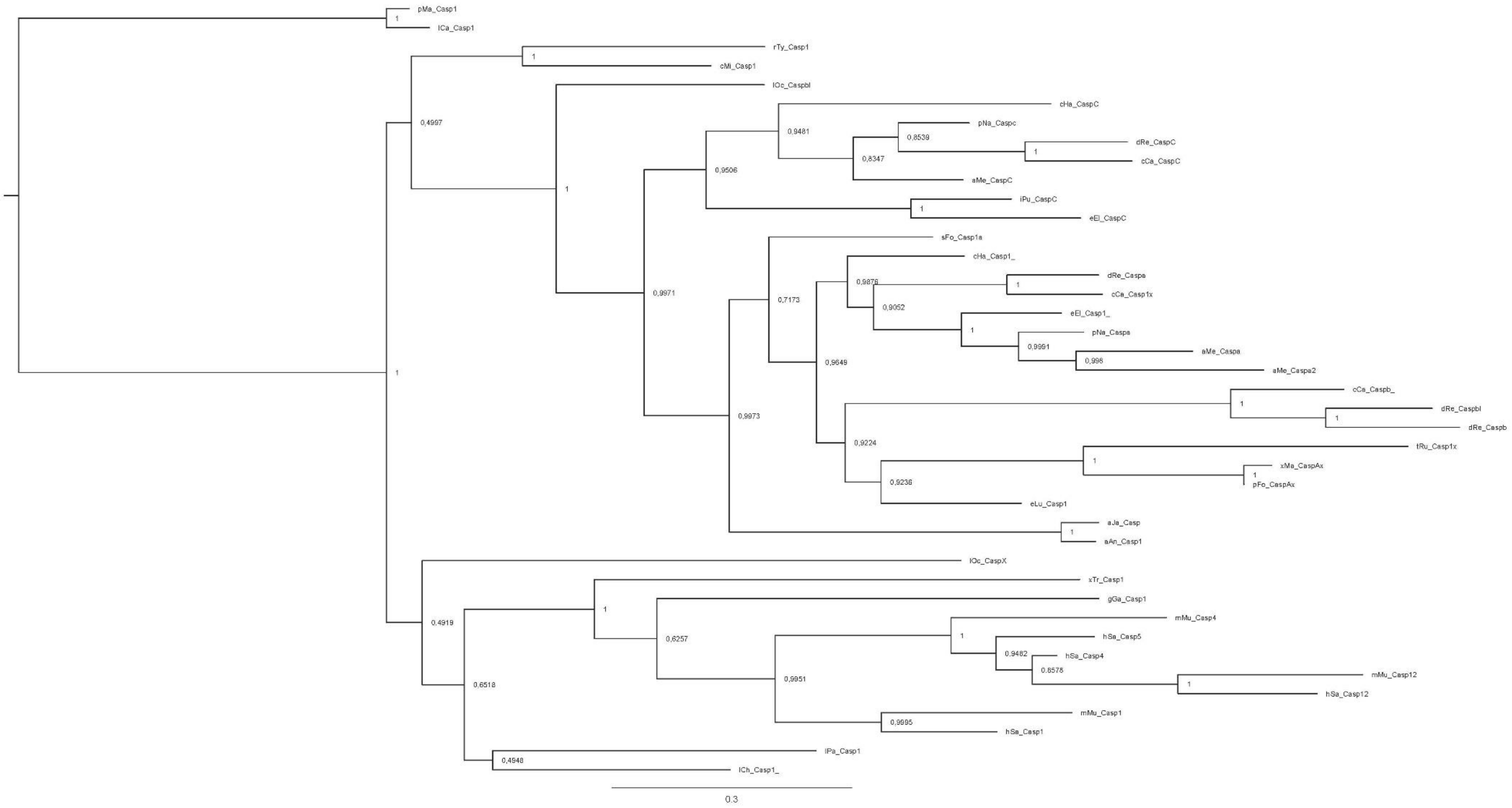

Figure 2. Maximum Likelihood gene tree calculated by Phyml. Node labels indicate statistical confidence.

Our evolutionary model resolves the origin and extent of the different prodomains in fish caspase-1-like: with the exception of the Otomorpha lineage, all caspase-1-like in fish with publicly available sequenced genome or transcriptome to date have a CARD prodomain. The first event in the Otomorpha lineage is the appearance of a prodomain-less caspase-1-like, the homolog of *caspc*. While the position of its cluster on the phylogenetic analysis is not accurately resolved, a conserved synteny within the all species of the Otomorpha lineage and the absence of a *caspc* ortholog in all currently available non-Otomorpha fish genomes and transcriptomes suggests that it emerged in this lineage (Figure S3).

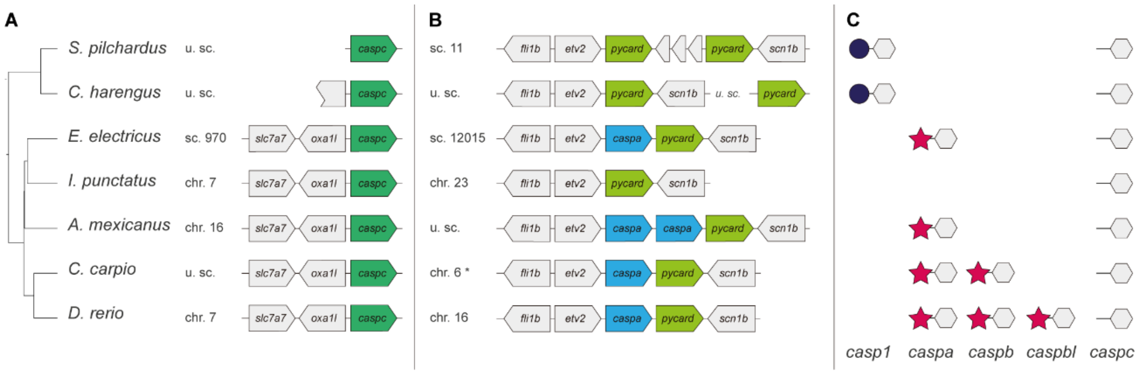

Figure S3. **A**) Conserved synteny of the *caspc* and **B**) the *pycard/caspa* locus in the Otomorpha lineage. **C**) Domain analysis of the caspase-1-like genes in the Otomorpha lineage. The CARD and PYD prodomains, and the catalytic domains are depicted with a blue circle, a red star, and a grey hexagon, respectively.

The model places later in the evolution, in the Otophysi lineage, the most notable difference between zebrafish and human proinflammatory caspases: the CARD to PYD prodomain swap. The lack of any caspase-1-like with CARD prodomain in the Otophysi lineage suggests a relocation of the caspase-1 evolutionary precursor next to *asc*, thus creating a fusion between the caspase catalytic domains and the *asc* PYD domain, or a caspase-1 gene duplication followed by a gene loss of the ancestral gene. Interestingly, a similar event in the caspase-1-like family evolution has been reported in the fusion of the neighbouring caspase-1 and caspase-4 genes in carnivores [14]. Tandem duplications of the *asc* gene seem common in fish species (e.g., *Astyanax mexicanus*, *Oryzias latipes*, *Gasterosteus aculeatus*), and can be found in the Clupea species *Sardina pilchardus*, potentially before the Otophysi divergence (Figure S3B). Consistent with these results, Otophysi species (e.g., *Electrophorus electricus*, *Ictalurus punctatus*, *Astyanax mexicanus*, *Pygocentrus nattereri*, *Cyprinus carpio*, *Danio rerio*) have PYD prodomains in their non-*caspc* caspase-1-like homologs, while non-Otophysi fish lineages (e.g., *Sardina pilchardus*, *Clupea harengus*, *Takifugu rubripes*, *Xiphophorus maculatus*, *Poecilia formosa*) retain the original CARD prodomain instead (Figure S3C).

Together with Caspa, a Caspb ortholog with PYD prodomain is present in cyprinids (e.g., *Cyprinus carpio*, *Carassius auratus*, *Ctenopharyngodon idella*), suggesting a gene duplication before the Cryprinidae lineage. Finally, phylogenetic and syntenic analysis suggests that Caspb-Caspbl is a recent zebrafish-specific tandem duplication.

Reference

1. Burge, C.; Karlin, S. Prediction of complete gene structures in human genomic DNA. *J. Mol. Biol.* **1997**, *268*, 78–94.
2. Marchler-Bauer, A.; Lu, S.; Anderson, J.B.; Chitsaz, F.; Derbyshire, M.K.; DeWeese-Scott, C.; Fong, J.H.; Geer, L.Y.; Geer, R.C.; Gonzales, N.R.; et al. CDD: A Conserved Domain Database for the functional annotation of proteins. *Nucleic Acids Res.* **2011**, *39*, D225–D229.
3. Sigrist, C.J.; de Castro, E.; Cerutti, L.; Cuche, B.A.; Hulo, N.; Bridge, A.; Bougueleret, L.; Xenarios, I. New and continuing developments at PROSITE. *Nucleic Acids Res.* **2013**, *41*, D344–D347.

4. Katoh, K.; Kuma, K.; Toh, H.; Miyata, T. MAFFT version 5: Improvement in accuracy of multiple sequence alignment. *Nucleic Acids Res.* **2005**, *33*, 511–518.
5. Talavera, G.; Castresana, J. Improvement of Phylogenies after Removing Divergent and Ambiguously Aligned Blocks from Protein Sequence Alignments. *Syst. Biol.* **2007**, *56*, 564–577.
6. Darriba, D.; Taboada, G.L.; Doallo, R.; Posada, D. ProtTest 3: Fast selection of best-fit models of protein evolution. *Bioinformatics* **2011**, *27*, 1164–1165.
7. Page, R.; Charleston, M.A. From Gene to Organismal Phylogeny: Reconciled Trees and the Gene Tree/Species Tree Problem. *Mol. Phylogenetics Evol.* **1997**, *7*, 231–240.
8. Hedges, B.S.; Dudley, J.; Kumar, S. TimeTree: A public knowledge-base of divergence times among organisms. *Bioinformatics* **2006**, *22*, 2971–2972.
9. Sjöstrand, J.; Sennblad, B.; Arvestad, L.; Lagergren, J. DLRS: Gene tree evolution in light of a species tree. *Bioinformatics* **2012**, *28*, 2994–2995.
10. Ronquist, F.; Teslenko, M.; van der Mark, P.; Ayres, D.L.; Darling, A.; Höhna, S.; Larget, B.; Liu, L.; Suchard, M.A.; Huelsenbeck, J.P. MrBayes 3.2: Efficient Bayesian Phylogenetic Inference and Model Choice Across a Large Model Space. *Syst. Biol.* **2012**, *61*, 539–542.
11. Guindon, S.; Dufayard, J.-F.; Lefort, V.; Anisimova, M.; Hordijk, W.; Gascuel, O. New Algorithms and Methods to Estimate Maximum-Likelihood Phylogenies: Assessing the Performance of PhyML 3.0. *Syst. Biol.* **2010**, *59*, 307–321.
12. Vernot, B.; Stolzer, M.; Goldman, A.; Durand, D. Reconciliation with Non-Binary Species Trees. *J. Comput. Biol.* **2008**, *15*, 981–1006.
13. Sennblad, B.; Schreil, E.; Sonnhammer, A.-C.; Lagergren, J.; Arvestad, L. primetv: A viewer for reconciled trees. *BMC Bioinform.* **2007**, *8*, 148.
14. Eckhart, L.; Ballaun, C.; Hermann, M.; VandeBerg, J.L.; Sipos, W.; Uthman, A.; Fischer, H.; Tschachler, E. Identification of Novel Mammalian Caspases Reveals an Important Role of Gene Loss in Shaping the Human Caspase Repertoire. *Mol. Biol. Evol.* **2008**, *25*, 831–841.
